# Do cognitive load and ADHD traits affect the tendency to prioritise social information in scenes?

**DOI:** 10.1177/17470218211066475

**Published:** 2022-01-04

**Authors:** Astrid Priscilla Martinez-Cedillo, Kevin Dent, Tom Foulsham

**Affiliations:** Department of Psychology, University of Essex, Colchester, UK

**Keywords:** Cognitive load, working memory, salience, social information, ADHD traits

## Abstract

We report two experiments investigating the effect of working memory (WM) load on selective attention. Experiment 1 was a modified version of Lavie et al. and confirmed that increasing memory load disrupted performance in the classic flanker task. Experiment 2 used the same manipulation of WM load to probe attention during the viewing of complex scenes while also investigating individual differences in attention deficit hyperactivity disorder (ADHD) traits. In the image-viewing task, we measured the degree to which fixations targeted each of two crucial objects: (1) a social object (a person in the scene) and (2) a non-social object of higher or lower physical salience. We compared the extent to which increasing WM load would change the pattern of viewing of the physically salient and socially salient objects. If attending to the social item requires greater default voluntary top-down resources, then the viewing of social objects should show stronger modulation by WM load compared with viewing of physically salient objects. The results showed that the social object was fixated to a greater degree than the other object (regardless of physical salience). Increased salience drew fixations away from the background leading to slightly increased fixations on the non-social object, without changing fixations on the social object. Increased levels of ADHD-like traits were associated with fewer fixations on the social object, but only in the high-salient, low-load condition. Importantly, WM load did not affect the number of fixations on the social object. Such findings suggest rather surprisingly that attending to a social area in complex stimuli is not dependent on the availability of voluntary top-down resources.

## Introduction

Previous research has focused on how visual attention and working memory (WM) interact in the context of distractor interference (Cashdollar et al., 2013; Downing, 2000; Konstantinou & Lavie, 2020; Lavie, 2010; Olivers et al., 2006). When shopping in the supermarket for a particular product, you must retrieve information from long-term memory about the appearance of the target and hold it in your WM, creating a target template (e.g., Bundesen, 1990; Duncan & Humphreys, 1989). This target template serves to specify your goal during the shopping expedition and should serve to guide your attention towards the sought-after product. However, the supermarket is filled with competing products that you do not intend to purchase. To choose the target product, it is important to avoid these distractors. Avoiding interference from irrelevant distractors can be especially difficult when they are physically salient (recall the bright red packaging of the Doritos pack). It seems likely that under such a scenario, increasing our cognitive load by trying to remember the phone number for the taxi we need to call to return home will increase the interference from these highly salient distractor products and prolong our shopping trip.

The load theory of attention and cognitive control provides a concrete framework that captures the links between visual attention and WM (Forster et al., 2014; Konstantinou & Lavie, 2020; Lavie, 2005, 2010; Lavie et al., 2004). Load theory proposes that an increase in the perceptual difficulty of a primary task (perceptual load) serves to reduce the perceptual processing resources available to process task irrelevant distractors, thereby reducing the extent to which these distractors interfere (Konstantinou & Lavie, 2020; Lavie et al., 2004). In addition, disrupting the availability of WM resources to maintain our goals serves to increase interference from task irrelevant distractors (Cashdollar et al., 2013; Forster et al., 2014; Lavie, 2010; Lavie et al., 2004). Relatedly, the executive attention theory proposes that WM capacity varies between subjects and between different cognitive tasks, as a consequence of executive-control processes involved in storing and retrieving stimuli in the face of conflict or distractors (Engle, 2002; Poole & Kane, 2009). This theory suggests that greater WM capacity entails enhanced filtering of irrelevant distractors (Poole & Kane, 2009).

Consistent with such theories, behavioural experiments demonstrate a crucial role for WM in modulating distractor interference. In the flanker task (e.g., Eriksen & Eriksen, 1974), when participants attempt to select a target while ignoring a distractor, performance is slowed when an irrelevant distractor is incompatible with the target (e.g., *x* when the target was *z*), and this interference increases under high WM load (Forster et al., 2014; Lavie, 2005; Lavie et al., 2004). Interference from a physically salient distractor has also been shown to increase under a high WM load (Lavie & De Fockert, 2005). These results demonstrate that the ability to reject distractors is impaired when WM is taxed, suggesting that WM plays an important role in attentional selection. Recently, there has also been evidence that eye movements reflect WM load during scene viewing. In particular, fewer fixations are made when participants are required to hold information in memory, compared with when they are unencumbered (Cronin et al., 2020). However, it remains to be seen whether guidance to specific items (i.e., the decision of “what” to look at) is affected by WM load in complex images. The primary aim of this study was to investigate whether loading WM would interfere with the default preference to look at specific areas in scenes.

### What determines where people look in scenes?

The physical properties of stimuli can be an important determinant of eye movements. In particular, previous research has identified salience from feature contrast (the extent to which an object differs from its surroundings) as a major determinant of interference (Itti & Koch, 2000; Theeuwes, 2010; Underwood et al., 2006; van Zoest & Donk, 2005; van Zoest et al., 2004). In research using simple displays, the presence of a singleton distractor (e.g., red distractor among green distractors) can cause significant interference with the ability to select a target (square among circles) (Theeuwes et al., 2003; Theeuwes & Failing, 2020). Such singleton capture can impact eye movements, in particular early fixations made within a few hundred milliseconds of viewing (Donk & van Zoest, 2008; van Zoest & Donk, 2005). Singleton interference also increases under a WM load (Lavie & De Fockert, 2005).

Other research has investigated the influence of stimulus salience in more complex scenes by comparing the observed pattern of fixations with those predicted by salience map models (Anderson et al., 2015; Foulsham & Underwood, 2007, 2008, 2009; Underwood et al., 2006). For instance, the Itti and Koch (2000) model suggests that each location in a scene is assigned a value that determines the likelihood that it will be fixated first. Across a set of basic feature dimensions (e.g., intensity, colour, and orientation) each object is compared with the local surround. Objects are more salient if they are locally distinctive, differing from the surround. Although it has been suggested that early fixations are made to salient regions (Anderson et al., 2015), the salience effect is strongly modulated by task instructions and demands (Foulsham & Underwood, 2007, 2008, 2009; Underwood et al., 2006).

Other studies have reported a more pervasive influence of socially relevant stimuli (e.g., people and faces within the picture) on eye movements (End & Gamer, 2019; Flechsenhar et al., 2018; Foulsham et al., 2010). In contrast to physical salience, social salience appears to bias both earlier and later fixations (End & Gamer, 2019; Flechsenhar et al., 2018). For example, End and Gamer (2019) found that when participants viewed naturalistic scenes, fixations were preferentially directed towards the heads of people appearing in the scene over areas that were merely high in physical salience, a preference that was similar regardless of whether participants were instructed to look at specific regions or not. Laidlaw et al. (2012) investigated how easy it would be for participants to avoid looking at specific areas of a face. Participants found it more difficult to avoid looking at the eyes than the mouth, but only when faces were upright, and not when they were inverted. Thus, it appears that the bias towards specifically social stimuli may be strongly automatic in the sense that it is obligatory and difficult to voluntarily override. The goal of this study was to further investigate the social bias in scene viewing by testing whether the bias to view social objects is dependent on top-down control resources.

We have reviewed how our eye movements might be guided by both bottom-up physical salience and top-down mechanisms, and how attention might be disrupted when WM is loaded with information. In this study (Experiment 2), we used complex pictorial stimuli which included a social object and a non-social object with known bottom-up visual salience. Previous studies on image-viewing have demonstrated how our attention is guided by top-down knowledge when we search for a specific known target (i.e., during visual search: Foulsham & Underwood, 2007, 2008, 2009; Underwood et al., 2006). However, top-down knowledge may be less important during free-viewing when there is no explicit target. We asked whether any tendency to preferentially view socially meaningful objects (e.g., people) over salient but non-social items would be disrupted by a WM load during free-viewing. Here, we investigate attentional guidance in the presence of load, by examining the time course of eye-movement behaviour when facing social and non-social objects with high and low salience.

### Individual differences in image-viewing

We also consider whether individual differences might affect the interactions between WM load, top-down, and bottom-up visual attention. Recently, Hayes and Henderson (2017, 2018) investigated how scan patterns during scene viewing are related to individual differences in intelligence, WM capacity, and speed of processing. Participants with higher and lower WM spans showed systematic differences in fixation patterns. Specifically, participants with the highest scores tended to fixate more on the top left-hand side of the image (Hayes & Henderson, 2017). Hayes and Henderson (2018) also investigated individual differences in traits associated with several disorders of cognitive processing: attention deficit hyperactivity disorder (ADHD), autism spectrum disorder (ASD), and dyslexia. These traits were assessed by self-report questionnaires in normal individuals (i.e., without a clinical diagnosis). Both ASD and ADHD traits were associated with some specific spatial patterns (e.g., a tendency to fixate the upper half of the image). Given these findings, it appears that particular patterns of scanning behaviour may be associated with individual differences in clinically relevant cognitive traits. However, it is not clear yet whether these individual differences affect looking at particular salient or social objects.

In this study, we focus on individual differences related to ADHD, a disorder with an overall population prevalence of 5.29% worldwide (American Psychiatric Association, 2013). While primarily a disorder affecting children, it can persist into adulthood, albeit with reduced prevalence of 2.5%–4% of adults (American Psychiatric Association, 2013; Faraone, 2000). ADHD is a heterogeneous disorder in which clinical diagnosis is associated with deficits in visual attention, WM, and inhibition (Barkley, 1997; Faraone, 2000; Nigg, 2001; Sergeant et al., 2003). In the past decade, extensive research has been devoted to the study of clinical-like traits within subclinical non-diagnosed community samples (i.e., Crosbie et al., 2013) or with unaffected siblings (Gau & Shang, 2010; van Ewijk et al., 2014). Typically, ADHD diagnosed individuals perform worse than those without ADHD at WM tasks (Gau & Shang, 2010; Kasper et al., 2012; van Ewijk et al., 2014). However, there are inconsistent findings regarding similar deficits in subclinical populations. Whereas Gau and Shang (2010) reported that unaffected siblings’ WM ability was as impaired as the clinical group, van Ewijk et al. (2014) reported that unaffected siblings’ WM ability was unimpaired. Research has reported that boys diagnosed with ADHD made slower and less accurate saccades than their typical counterparts in a search task (Van der Stigchel et al., 2007). In addition, children diagnosed with ADHD are reported to have a poorer ability to maintain fixation at a fixed position in comparison with a typical group (Caldani et al., 2019). Research has also shown that people with high but subclinical levels of ADHD-like traits have an abnormal rate of microsaccades in comparison with those with lower levels of ADHD-like traits in a sustained fixation task (Panagiotidi et al., 2017). Of particular relevance to the current work, participants with clinically diagnosed ADHD showed increased interference from an irrelevant distractor in comparison with healthy controls (Forster et al., 2014). Together, these findings indicate impairments in both WM mechanisms and distractor rejection in those clinically diagnosed with ADHD as well as some evidence of atypical eye movements in those with ADHD-like traits.

### The current study

The main aim of this study was to determine the role of top-down control processes related to WM in determining viewing patterns in complex images. To this end we investigated how maintaining a high or low memory load would impact viewing patterns. According to the load theory of selective attention, (Cashdollar et al., 2013; Forster et al., 2014; Lavie, 2010; Lavie et al., 2004), high WM load should disrupt top-down cognitive control. If the bias to look at social stimuli arises as a consequence of top-down goals, we should expect this bias to be reduced under conditions of high WM load. This might especially be the case in the face of strong, bottom-up physically salient objects in the scene. In contrast if the bias towards socially salient stimuli arises in a way independent of top-down mechanisms related to WM it should be unimpeded (End & Gamer, 2019; Flechsenhar et al., 2018). Importantly, this study used a verbal memory load as a means to create a cognitive load, interfering with domain general processes of cognitive control. A visuo-spatial memory load was specifically avoided since previous research (e.g., Konstantinou & Lavie, 2020) demonstrates that such tasks may also increase the overall perceptual load by competing with the task relevant processes for perceptual resources. As the aim of this study was specifically to investigate cognitive load, a verbal memory task with relatively little draw on visual perceptual processing resources was chosen.

Furthermore, we aimed at studying whether the severity of subclinical symptoms of ADHD might affect eye movements while free-viewing the scenes. If the tendency to select scene objects depends on top-down control processes linked to WM and these processes are impaired in those displaying ADHD behaviours (Crosbie et al., 2013; Forster et al., 2014; Gau & Shang, 2010; Kasper et al., 2012; van Ewijk et al., 2014), increased ADHD traits may serve to reduce the bias towards socially relevant objects.

In the experiments reported here, we first verified that our manipulation of memory load was adequate to disrupt performance in the classic flanker (Eriksen & Eriksen, 1974) task (a replication of Lavie et al., 2004, Experiment 1). Experiment 2 then examined how the pattern of eye movements that participants make while free-viewing complex images would be affected by the same memory load manipulation. We measured natural-looking behaviour, simply asking participants to freely view the images with no specific task. The scene stimuli contained multiple objects, one of which was a critical “social object”. In addition, in each scene a non-social object was identified, and two versions of the scene were created. In the low physical salience condition the object was unchanged, whereas in the high physical salience version the object was edited in a way to increase its physical salience (estimated using Itti & Koch’s (2000) model). We expected to find a preference for the social object even in the presence of a physically salient object, as has been demonstrated previously (Birmingham et al., 2009; End & Gamer, 2019). Our major research questions were how this bias would be affected by a WM load and by ADHD-like traits.

## Experiment 1

The purpose of Experiment 1 was to confirm that the specific implementation of WM load used in this study can affect attentional selection, in line with previous studies (Lavie et al., 2004). It was important to verify the effectiveness of our manipulation of WM before employing it in a novel context in Experiment 2.

### Method

#### Participants

Twenty-one students from the University of Essex participated. We aimed for a sample size greater than that in the original study (Lavie et al., 2004, Experiment 1; 11 participants). We also carried out a power analysis by simulation (using Superpower; Lakens & Caldwell, 2021, and assuming a strong within-subjects correlation). This indicated that even a sample of 5 participants is enough to detect the original main effect of compatibility, and that a sample of 18 participants results in good power for the interaction (both at 80% power).

All of the subjects reported normal or corrected-to-normal vision. Participants were paid £4 or 1 credit for their participation. The study was approved by the ethics board of the University of Essex.

#### Task and stimuli

**Figure 1.**
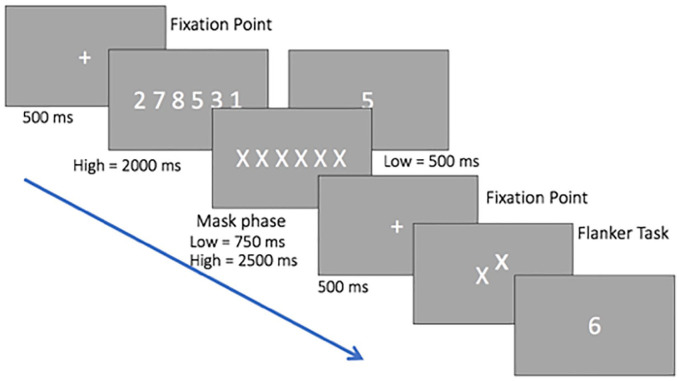
Schematic representation of the stimuli and procedure of Experiment 1. Digits are shown larger than in the actual experiment.

The experiment was programmed in MATLAB (Version 9.1.0, R2016b; the MathWorks, Natick, MA), using the Psychophysics Toolbox. We replicated Experiment 1 from Lavie et al. (2004), in which participants performed a selective attention task (a flanker task: Eriksen & Eriksen, 1974) while simultaneously performing a WM task. Figure 1 illustrates the procedure for Experiment 1. Each trial started with a fixation dot displayed for 500 ms, followed by the WM load display. For the one-digit presentation (low load) this remained on the screen for 500 ms and for the six-digit presentation (high load) for 2,000 ms. For both loads the digits were chosen randomly from 1 to 9, with no repetition and in a random order. A mask display was then presented for 750 ms for the one-digit presentation and 2,500 ms for the six-digit presentation, followed by a fixation point presented for 500 ms. The target letter in the selective attention task was either a *z* or an *x*, presented in lowercase and located in the centre of the screen. A distractor letter (the flanker) was presented above or below the target and was either compatible (i.e., *x–x*), incompatible (i.e., *x–z*) or neutral (i.e., the letter *n*). For the selective attention task, participants were required to press *z* if the target letter on the display was a *z*, or *x* if the target letter on the display was an *x*. After the response to the selective attention task, participants saw a probe digit and were required to respond whether this was presented previously by pressing the right or left arrow key on the keyboard. Participants were instructed to respond as fast as possible in both tasks. All the combinations (target identity, distractor identity, and distractor position) were counterbalanced and presented in a random order. According to these specifications, 90 displays were created for each condition of WM load. Load conditions were blocked and presented in a counterbalanced order between participants. The total duration of the experiment was approximately 40 min.

### Data analysis

Only participants who scored above chance on both tasks were included in the analysis. This resulted in five exclusions. From the remaining 16 participants’ data, only trials on which the participants were correct in the memory task and with reaction times (RTs) over 100 ms and under 2,000 ms were included in the analysis. On average, participants had 166.5 (*SD* = 10.45) trials remaining after exclusions.

### Results

Accuracy in the memory probe was lower in the high-load condition (*M* = 91.82%, *SD* = 7.57), and slightly higher in the low-load condition (*M* = 95.90, *SD* = 4.78).

Table 1 presents the mean and standard deviation RT on the flanker task as a function of WM load and distractor compatibility. A two-way within-subject analyses of variance (ANOVAs) on flanker RT as a function of WM load (low, high) and distractor compatibility (compatible, incompatible) revealed a significant main effect for distractor compatibility *F* (1, 15) = 18.484, *p* = .001, η² = .552, indicating that responses in the compatible condition are significantly faster than the incompatible condition. There was no significant main effect of memory load on RTs *F* (1, 15) = 2.195, *p* = .159, η² = .128. However, there was a significant interaction between WM load and distractor compatibility *F* (1, 15) = 7.897, *p* = .013, η² = .345. Follow-up, paired comparisons revealed that distractor compatibility effects (compatible vs incompatible) were significant in high-load trials, *t* (15) = −5.405, *p* < .001, but reduced such that they failed to reach significance in low-load trials, *t*(15) = −1.852, *p* = .08.

**Table 1.** Mean correct reaction times (in milliseconds) on the flanker task as a function of the WM and distractor compatibility.

WM	Low		High	
	*M*	*SD*	*M*	*SD*
Compatible	946	126	960	106
Incompatible	992	136	1,083	122
Neutral	994	162	1,061	167

WM: working memory.

This experiment confirms that the manipulation of memory load was adequate to disrupt performance in a response competition task which is consistent with the load theory of selective attention (Lavie et al., 2004).

## Experiment 2

Experiment 2 applied the WM manipulation used in Experiment 1 to an image-viewing task to examine how viewing patterns might change as a function of WM load. Looking around an image requires moment-by-moment decisions about where to place the eyes, and these decisions can be thought of as a competition between different potential targets for attention (Foulsham, 2019). The results of Experiment 1 (and the load theory of selective attention) indicate that attending to targets and avoiding distractors is more difficult in conditions of high load. In Experiment 2, we measured fixations to each of two objects: (1) a social object and (2) a non-social object with high or low salience. As we expected the social object to attract more attention, we can think of the non-social object as akin to a flanking distractor. Furthermore, we examined the relationship between individual differences in clinical traits of ADHD in a community sample and overall performance on the task. Hence, we considered the probability of fixating on each object (social and non-social) with high and low saliency, performance in the WM task in both loads (high and low), and the scores from the ADHD-like symptoms by using the Adult ADHD Self-Report Scale (ASRS) questionnaire (Kessler et al., 2005).

### Method

#### Participants

We tested 60 participants in line with our pre-registration. The participants (aged 18–35, *M* = 24.28 years, 41 females) were recruited from the University of Essex. All participants reported normal or corrected to normal visual acuity. After discarding data from 10 participants who were not accurate in the calibration (above 0.8°, a threshold set a priori), the final sample consisted of 50. They were granted with £3 for their participation or course credits and were naïve of the purposes of the experiment. The study was approved by the ethics board of the University of Essex.

#### Apparatus and stimuli

The experiment was programmed in MATLAB (version 9.1.0, R2016b; the MathWorks, Natick, MA), using the Psychophysics Toolbox. Eye position was recorded using the SMI RED500, which is a screen-based eye tracker that samples pupil position at 500 Hz. A 9-point calibration and validation were repeated several times to ensure that all recordings had a mean spatial error of better than 0.8°. Head movements were restricted using a chin rest. The experiments took place in a dimly illuminated, sound-attenuated room. Participants sat 60 cm away from the monitor so that the stimuli subtended approximately 43° × 28° of visual angle at 1680 × 1050 pixels. A set of 64, high-resolution colour photographs were prepared as stimuli. Thirty-two pictures were used as fillers and the rest were selected following the criteria that they contained a person and an object on opposite sides of the image. The fillers were naturalistic scenes without a social element. They were included to avoid participants from noticing the structure and key objects in the experimental pictures.

All pictures were found from different free access image databases (e.g., Pixabay)

Before the experiment, participants were required to complete the ASRS (Kessler et al., 2005). This questionnaire consists of 18 symptoms related to the *Diagnostic and Statistical Manual of Mental Disorders*; 4th ed., text rev. (*DSM*-IV-TR) criteria for ADHD. Participants reported the frequency of the symptoms experienced over the past 6 months. The questionnaire uses a 5-point Likert-type scale which spans 0 for *never*, 1 for *rarely*, 2 for *sometimes*, 3 for *often*, and 4 for *very often*. Participants were given verbal and written instructions regarding the experimental procedures.

**Figure 2.**
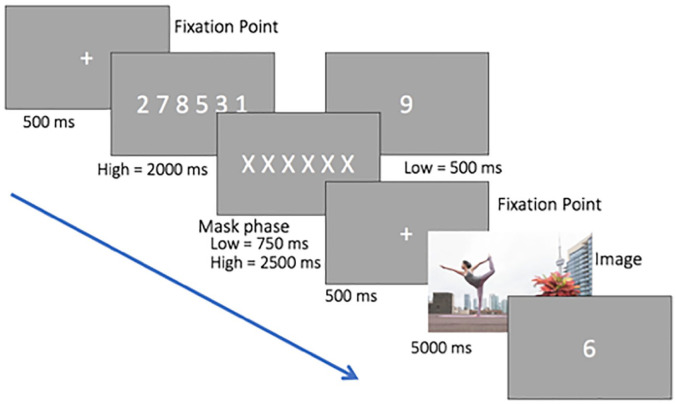
Schematic representation of the stimuli and procedure of Experiment 2. This condition is high WM and features a high salient non-social object. Digits are shown larger than in the actual experiment.

Figure 2 illustrates the procedure for each trial in Experiment 2. Calibration and validation of the eye tracker was performed at the start of each session. The memory task was the same as in Experiment 1. In the image-viewing task, the picture was shown for 5,000 ms. Participants were instructed to look freely at the picture. After the scene, the memory probe display was presented. Participants were required to respond whether the probe digit was presented previously by pressing the right or left arrow key on the keyboard.

The experiment consisted of two blocks: one-digit (low load) and six-digit (high load) presentation. Each block consisted of 32 trials, which included 16 experimental pictures and 16 fillers, randomly intermixed. Half of the participants started with the one-digit block and the other half with the six-digit block. Experimental images were counterbalanced across participants such that each particular scene appeared in all load and salience conditions, and each was mirror reversed for half the participants to control for any biases to the left or right of the image. There were a total of eight different versions formed by a combination of the following factors: flipped image (original, flipped), memory probe (present or absent), and object salience (high or low). Participants were assigned randomly to one of the eight different versions. Only the factors of distractor salience and memory load were of theoretical interest. The experiment took a total of approximately 25 min.

#### Salience maps for each non-social object within the image

The 32 experimental pictures were edited to change the salience of the non-social object. We checked the salience of these regions using the Saliency Toolbox (Walther & Koch, 2006) via MATLAB (version 9.1.0, R2016b; the MathWorks, Natick, MA) before and after a change. The salience of the non-social object was classified based on the first three simulated fixations. In half of the pictures, this object was classified as highly salient because it received one of the first three simulated fixations. The other 16 pictures were classified as containing a low salience object which was not selected until later simulated fixations. Classifying region salience in this way is an alternative to analysing the values in the salience map which does not require assumptions about how the map is normalised, but both methods produce similar results (see Foulsham, 2005; Foulsham, 2019; Foulsham & Underwood, 2007). We used PicMonkey to increase and/or decrease the salience of each object within the image as well as incorporating an object to some stimuli that did not contain any. In practice, object salience was modified by changing the colour or luminance to increase or decrease the contrast relative to the background. As described above, all images were flipped for half the participants to ensure that object type and salience were not confounded with spatial position. The social object was a person, of which there was only one in each image. The social object was never one of the three most salient locations in the scene. The non-social object was chosen from one of the bigger or more prominent inanimate objects in the scene.

**Figure 3.**
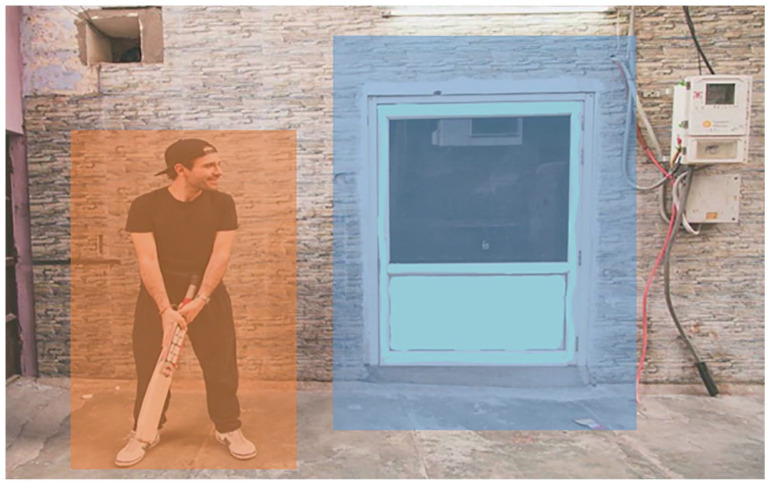
An example of one scene from Experiment 2, with a high-salient non-social object. This figure also shows an example of the regions of interest (ROI) for a target stimulus. Note the squares delineating the ROI were not visible during the experiment.

Figure 3 depicts one image as presented in the high-salience condition. The social region of interest (ROI) is the man. The non-social object is the door frame.

### Data analysis

Participants who scored below 50% on the memory probe were excluded from the analysis. Fixations were removed if their duration was below 100 ms. We also excluded trials where the starting fixation was not recorded on the centre and those with incorrect memory responses. Following these criteria, we analysed data from 45 participants. Power simulations (Superpower; Lakens & Caldwell, 2021) indicated that this sample size with a 2 × 2 within-subjects design produces excellent power for detecting even small main effects, as well as moderate interactions, even with a small within-subjects correlation. We delineated an ROI around each social or non-social object to enable analysis of fixations (for an example see Figure 3). On average, the social object ROI covered 23% of the image area, while the non-social object covered 20.50% of the image area. Across the images, this was not a significant difference, paired *t*-test, *t*(31) = −1.111, *p* = .275.

### Results

We examined the effect of WM load on the image-viewing task. Our analysis was based on the two ROIs: social and non-social, described above. We first examined the effect of WM load on fixations to both ROIs (social and non-social). Then, we examined the effect of WM load and salience on fixations to the non-social ROI. Finally, we investigated whether symptoms of ADHD are related to eye-movement behaviour as well as accuracy and RT in the memory task. Our dependent variables were (1) accuracy in the WM task, (2) RT in the WM task, (3) total number of fixations, (4) average fixation duration per ROI, (5) overall probability of fixations on the non-social object, (6) overall probability of fixations on the social object, and (7) the ADHD trait scores from the ASRS.

#### Behavioural data

Accuracy in the memory task was slightly lower in the high-load condition (*M* = 88.52%, *SD* = 12.66) than in the low-load condition (*M* = 94.50%, *SD* = 9.09). A paired sample *t*-test was conducted to compare the RT with the memory probe under high and low loads. The RT in high-load trials (*M* = 1,447 ms, *SD* = 1,064) and low-load trials (*M* = 1,149 ms, *SD* = 642) was only marginally different, although again this difference was consistent with the high-load condition being more difficult; *t* (44) = −1.840, *p* = .072.

#### General eye-movement statistics

Figure 5 shows an example of the fixation locations made by one participant during the task. In the example scene, the participant made a greater number of fixations on the social object and fewer on the non-social low salient object. Table 2 shows general eye-movement statistics across conditions and across participants as a function of WM load and salience to the non-social object. We analysed the number of fixations to get an overall idea of viewing behaviour as well as the mean duration of fixations. There was no reliable effect of memory load, *F* (1, 44) = .016, *p* = .899, η² < .001, or salience, *F*(1, 44) = .854, *p* = .361, η² = .019, on average fixation duration and no interaction of load and salience *F*(1, 44) = .542, *p* = .466, η² = .012. There was also no effect of load, *F*(1, 44) = 2.062, *p* = .158, η² = .045, or salience, *F*(1, 44) = .944 *p* = .337, η² = .021, on fixation count and no interaction of memory and salience *F*(1, 44) = .346, *p* = .559, η² = .008.

**Table 2.** The total number and average duration of fixations per participant as a function of condition.

WM	High load		Low load	
Saliency of non-social object	HS	LS	HS	LS
*N* fixations				
*M*	56.64	59.91	63.00	63.80
*SD*	33.72	33.04	29.82	26.91
Average fixation duration in milliseconds				
*M*	316.37	320.45	313.13	325.67
*SD*	80.53	69.52	78.19	59.75

WM: working memory; HS: high salience; LS: low salience.

#### The effect of WM load on fixations to the high- and low-salient non-social object

We first considered the proportion of fixations on the non-social object (see Table 3). Participant means were entered into a within-subject ANOVA with the factors of memory load (high and low) and non-social object salience (high and low). There was a significant effect of salience, *F*(1, 44) = 4.565, *p* = .038, η² = .094, indicating that participants looked more often at the higher salience object. There was a trend towards an effect of memory load, *F*(1, 44) = 2.967, *p* = .092, η² = .063, with slightly more fixations on the non-social object during the high-load condition. However, there was no interaction between memory load and object salience, *F*(1, 44) = .284, *p* = .597, η² = .006. Thus, participants looked more at the non-social object when it was higher in salience, regardless of the memory load.

**Table 3.** The percentage of fixations on each region of interest: social and non-social object.

WM	High load		Low load	
Saliency of non-social object	HS	LS	HS	LS
ROI	Non-social object			
*M*	27.41%	21.96%	23.78%	20.22%
*SD*	13.12%	15.37%	11.32%	12.43%
ROI	Social object			
*M*	41.54%	41.74%	41.82%	41.84%
*SD*	13.37%	16.52%	14.92%	18.17%

WM: working memory; HS: high salience; LS: low salience; ROI: region of interest.

A second analysis was performed on the proportion of fixations to the social object. Participant means were entered into a within-subject ANOVA with the factors of memory load (high and low) and non-social object salience (high and low). This revealed no effects of load *F* (1, 44) = .008, *p* = .931, η² = .000, or object salience *F* (1, 44) = .002, *p* = .966, η² = .000, and no interaction between load and object salience *F* (1, 44) = .002, *p* = .965, η² = .000, thus indicating that participants looked at the social area regardless of WM load and the salience of the competing non-social object. The percentages in Table 3 indicate that the social object was looked at more often than the non-social object, in all conditions.

**Figure 4.**
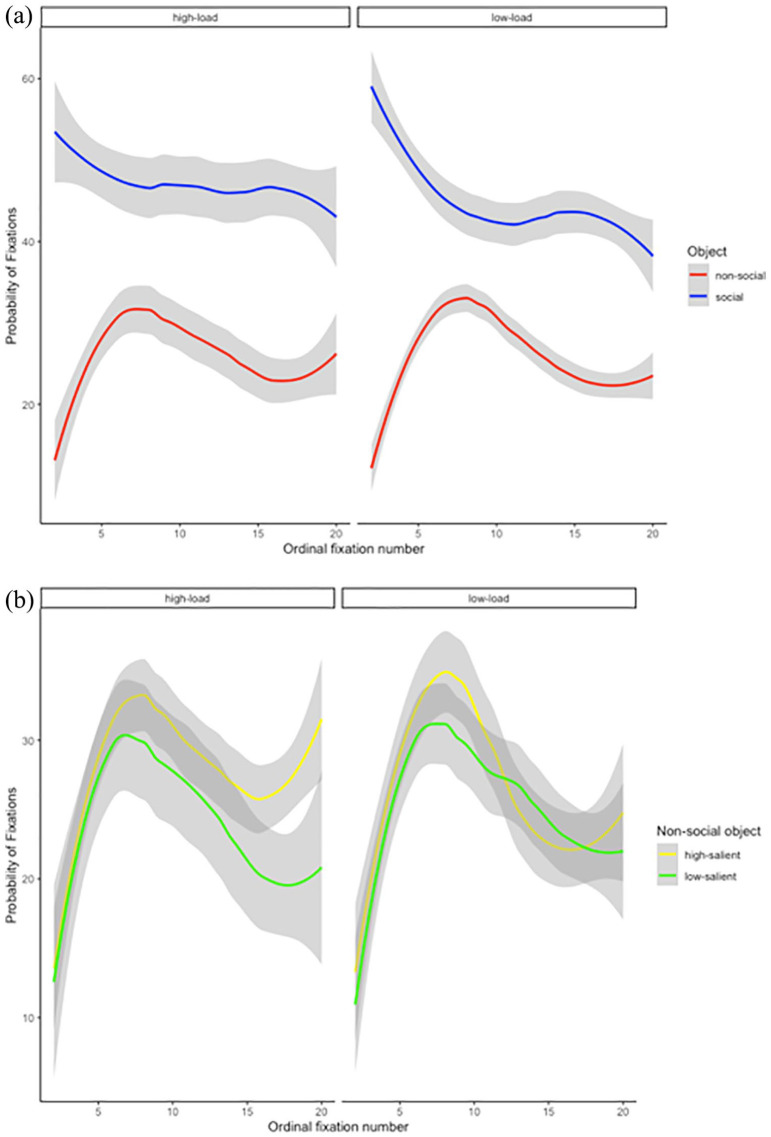
(a) The probability of fixations as a function of working memory load (high and low) and ROI (social and non-social). (b) The probability of fixations as a function of working memory load (high and low) and non-social object type (high salient and low salient). Note that ordinal fixation number begins at the second fixation, as the first fixation was at the centre of the scene. Lines represent the mean across participants with shading area representing the confidence interval. The *x*-axis is shown up until the 20th fixation; some trials would have gone longer.

When looking at the images, the viewers spent a greater number of fixations on the social object. Previous research has suggested that physical salience may have greater effects on the first few fixations, and we might expect the influence of top-down guidance and load to change over the course of viewing. To investigate this, we further calculated the probability of fixating on each ROI (social and non-social; see Figure 4a) and on the two types of non-social objects (high salience and low salience; see Figure 4b) as a function of WM load for each fixation number and participant. From the time course in Figure 4a, it is clear that fixations remain greater on the social region than on the non-social region, regardless of memory load, and that this advantage persists over time. From Figure 4b. it is clear that effects of salience are minor.

**Figure 5.**
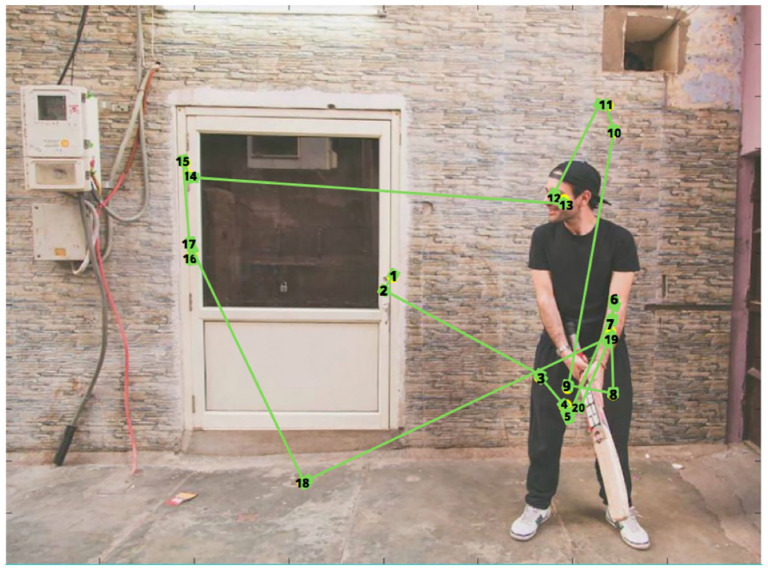
A visual representation of the locations fixated by one participant in the low WM load condition. Note that this scene is the same as Figure 2, but this image featured a low salient non-social object, and it is flipped. The numbers indicate ordinal fixation number. Fixations started at the centre of the picture, and attention is moved to the social object, followed by the non-social object.

#### The relationship between ADHD symptomatology and task performance

To examine whether our measures of attention in scenes were altered in those with high traits of ADHD, we correlated the total score of each participant from the ASRS questionnaire with the probability of fixations on the social area. Scores on the ASRS checklist varied from 12 to 49 and the mean score was 28.80 (*SD* = 8.27). Higher scores on the ASRS indicate higher levels of ADHD traits, although there is no clear clinical cut-off and diagnosis can be complex (Kessler et al., 2005). The correlation values are presented in Table 4. For most variables, the relationship was weak and non-significant. However, a weak relationship was found when correlating ADHD severity with probability of fixations on the social area. The direction shows that participants with higher scores in the ASRS questionnaire fixated less often to the social area, but this was only reliable in the low-memory and high-salient condition. There was also a suggestive correlation between RT to the memory probe and ASRS, but only in the high-load condition. This might indicate that those with ADHD traits found the WM task more difficult.

**Table 4.** Correlation values for ADHD severity and the fixation variables.

		Pearson’s *R* with ASRS score	*p* value
PF on social	High-load HS	−0.184	.226
	High-load LS	−0.104	.496
	Low-load HS	−**0.321**	**.031**
	Low-load LS	−0.111	.466
RT on correct responses	Low load	0.130	.396
	High load	0.263	.081

ADHD: attention-deficit/hyperactivity disorder ASRS: Adult ADHD Self-Report Scale; PF: probability of fixations; HS: high salience; LS: low salience; RT: reaction time. *N* = 45. Bold values represents significant values

## General discussion

Experiment 1 confirmed previous findings that increased memory load serves to increase distractor interference. Experiment 2 used an image-viewing task to examine how a WM load affects attention to social and non-social regions of interest. The images were modified to investigate the role of bottom-up physical salience. We also examined task performance related to ADHD traits. Since the memory load manipulation made a difference to the flanker task, we might expect it to also affect attention to different objects in Experiment 2. Specifically, the research reviewed in the “Introduction” led to the predictions that (1) increased WM load should disrupt top-down cognitive control, and therefore affect our viewing patterns; (2) our attention is biased to attend to social objects (other people) in complex settings (End & Gamer, 2019; Foulsham et al., 2010); (3) if the social bias is a consequence of default voluntary top-down goals, then it should be disrupted when memorising high loads of information; and (4) if object-selection depends on top-down processes which are impaired in ADHD (Barkley, 1997; Faraone, 2000; Nigg, 2001; van Ewijk et al., 2014), then higher traits of ADHD should lead to a reduced bias towards the social object.

Increasing salience biased the eye-movement patterns such that participants looked at the non-social object a little more when it was highly salient than when it was not. However, this small effect of physical salience was dwarfed by the very large effect of the social or non-social nature of the object being looked at. Our key research question was whether this bias would be affected by increasing WM load, and their answer was clear. WM did not change the overarching bias to spend more time looking at the social areas. Indeed, the tendency to fixate social areas was stable across conditions. This finding is compatible with the idea that such social biases stem from automatic processes which are relatively unaffected by load (End & Gamer, 2019; Foulsham et al., 2010; Laidlaw et al., 2012). The manipulation of salience on the non-social object had a small effect. A greater probability of fixations are likely to be on high-salient regions according to previous research, at least when there is no task requirement to look at anything else (Anderson et al., 2015; Foulsham & Underwood, 2007, 2008; Itti & Koch, 2000; Parkhurst et al., 2002; Underwood et al., 2006). Our results are consistent with the idea that bottom-up salience signals influence the control of attention. Although the high-salient object attracted attention, it does not seem to disrupt the bias to attend to social regions.

The social advantage is interesting given that participants were only asked to look freely around the image. One explanation of the social advantage is that participants have a preference to look at people (Crouzet, 2010; Di Giorgio et al., 2012; End & Gamer, 2019; Fletcher-Watson et al., 2008; Foulsham et al., 2010) in comparison to animals or objects (Crouzet, 2010; Fletcher-Watson et al., 2008). A very rapid bias towards images of people has been reported to emerge even 100 ms after stimulus presentation (Crouzet, 2010; Fletcher-Watson et al., 2008). Social areas may continue to hold our attention due to emotional and intentional information that can be obtained from looking at eyes or mouths (Birmingham et al., 2009; Foulsham et al., 2010). From an evolutionary perspective, monkeys and humans share a similar pattern of viewing behaviour to social objects (Guo, 2007; Guo et al., 2003; McFarland et al., 2013). Both look more to the face than the body area but attend more to the body area in a negative social context over a positive social context (McFarland et al., 2013). Both monkeys and humans are better at processing the eyes than other facial features (Guo, 2007; Guo et al., 2003). Such social prioritisation has also been reported in infants (Di Giorgio et al., 2012). Our data corroborate this social prioritisation even when cognitive resources are diverted to perform a secondary memory task.

It may seem surprising that participants in Experiment 2 were able to prioritise social information, even in the presence of a disruptive memory load (which, in Experiment 1, we demonstrated interfered with a basic flanker task). Social areas were more likely to be looked at, even on the first few fixations. It is possible that this rapid attention to faces, which does not seem to be disrupted by load, relies on “feedforward” processes which have been identified in cognitive neuroscience. Electroencephalogram (EEG) studies have reported face-responsive N170 brain activation occurring at even earliest latencies (i.e., Rossion et al., 2015). For instance, evidence shows brain activity between 120 and 400 ms after stimulus presentation that is initially widespread over the medial and lateral occipital cortices (Rossion et al., 2015). This phenomenon is also consistent with the findings of single cell studies in monkeys, which have reported that neurons in the inferotemporal cortex selective for faces have similar dynamic changes to those from the primary visual cortex, despite being conventionally activated much later in the hierarchy (Sugase et al., 1999). These neuron changes may reflect a feedforward sweep process whereby certain stimuli are processed quickly and boost “low-level” responses (Epshtein et al., 2008; Lamme & Roelfsema, 2000; Riesenhuber & Poggio, 1999; Sugase et al., 1999). This process may reflect pre-attentive vision, where the visual cortex is rapidly activated from low levels to high-level areas (Hochstein & Ahissar, 2002; Lamme & Roelfsema, 2000). In brief, social areas can generate feedback to lower hierarchical level before scenes are analysed in detail, thereby altering the subsequent sweep (Lamme & Roelfsema, 2000; Sugase et al., 1999).

In understanding our results, it is also useful to consider recent theoretical debates around attentional control and the meaning of the terms top-down and bottom-up (Benoni, 2018; Benoni & Ressler, 2020; Egeth, 2018; Gaspelin & Luck, 2018; Theeuwes, 2018). Some authors (e.g., Theeuwes, 2018) emphasise the importance of whether the control of attention is voluntary or involuntary, and argue from the existence of involuntary control of attention, that may occur despite our temporary goals to the contrary, that there are important limits to the influence of top-down goals on attentional control. Others (e.g., Benoni, 2018; Benoni & Ressler, 2020) argue that the control of attention is fundamentally driven by the relevance of the stimuli to our goals, but these goals are sometimes implicit such that we may not be aware of them, or deploy them deliberately. Benoni and Ressler (2020) suggest that by combining this implicit–explicit dimension, with a second dimension that captures the timescale over which a particular goal applies, most phenomena of attentional control can be explained. On this account, traditional forms of top-down control of attention where specific task-relevant goals are loaded into WM would be considered explicit and temporary. Returning to the current study the preferential looking towards the social object may best be characterised as the result of an enduring implicit goal. The current results are then consistent with the idea that the expression of such an enduring implicit goal can occur even in the face of a high cognitive load. The framework proposed by Benoni and Ressler (2020) may be useful in that it explains how both “low-level” physical and “high-level” social stimuli can influence attention according to fundamentally similar processes.

Our data suggest that in complex scenes, social objects dominate viewing patterns over salient objects, and they continue to do so even when memorising higher loads of information. This is perhaps surprising, as we might expect participants to try to avoid distraction while completing the memory task, for example, by looking only at the centre of the screen or avoiding meaningful regions. There was also no reliable effect of load on number of fixations or duration of fixations, although there were slightly fewer fixations in the high-load condition. This is a different pattern of results from Cronin et al. (2020), who reported effects of load on both number and duration of fixations, although this was more pronounced in a visual load than a verbal load condition. The finding that participants continue to look at people in the scene is in agreement with other research suggesting that attending to social information is rather automatic and hard to suppress (Laidlaw et al., 2012). That participants do not alter their natural fixation patterns while maintaining a large memory load, suggests that these task irrelevant fixations do not interfere with WM, or that attempting to override them would be more costly than allowing their natural expression.

In Experiment 1, WM load interacted with congruency in a flanker task. However, in Experiment 2, the very same memory load manipulation had little effect on fixation of a social object in the presence of other “distracting” objects. Given the results of Experiment 1 and previous studies, it seems unlikely that the levels of load used were not sufficiently difficult to produce interference, although future studies could try a more difficult task. The stimuli in Experiment 2 (images) were more complex than in Experiment 1 (single letters). However, the free-viewing task in Experiment 2 may have been too simple to result in a dual task situation comparable to the flanker task. This could be addressed in future studies by combining a memory load with an image-based task such as realistic visual search which explicitly requires scene processing. Importantly, it could also be that a different type of WM load would have more of an effect on guidance during picture viewing.

While the outcome of Experiment 1 demonstrates that our manipulation of cognitive load is effective in the context of the flanker task, the outcome of Experiment 2 remains a null result. As such, we must consider the possibility that other implementations of cognitive load may impact viewing patterns in scenes. We note that while we modelled our cognitive load task on that used by Lavie et al. (2004), a great variety of cognitive load tasks have been used in the literature. One issue here concerns the nature of the underlying cognitive mechanisms that subserve the memory task. Most models of WM (e.g., Baddeley, 2003; Baddeley & Hitch, 1974) distinguish between often domain-specific limited capacity storage of items, and other executive or control processes which serve to modulate and manipulate items held. Cognitive load tasks that have been used in the literature differ in the extent to which they require additional processes on top of the storage and retrieval of the items. For example, Burnham et al. (2014) showed that maintaining the phonological properties of nonsense syllables (e.g., gah, goo, gee) did not increase the magnitude of the interference from a salient distractor. However, counting backwards from a given starting point did serve to increase interference. These results suggest that tasks that tax executive-control processes in addition to the storage of the phonological properties of the items impose a greater cognitive load and modulate distractor interference to a greater extent. Our implementation of cognitive load is one that emphasises the storage of the phonology of the items, and it remains possible that other tasks with a greater executive demand may serve to disrupt viewing patterns in scenes.

In addition, in our task the low-load condition took the form of a one-item memory load, and this was compared against a six-item memory load in the high-load condition. In contrast frequently in the literature a high cognitive load condition is compared against a single task baseline (e.g., Burnham et al., 2014; Lavie & De Fockert, 2005, Experiment 1). The possibility remains that if a performance in the high cognitive load condition were compared against a single task baseline, a difference may be observed.

At the outset of the study, we reasoned that if social biases rely on a top-down process that is disrupted by the presence of ADHD-like traits, then a general disruption of the social biases across all conditions would be expected. However, this is not what we observed because high levels of ADHD-like traits were related to fewer fixation to the social object only in the low-load and high-salience condition. Any effects of ADHD traits in this experiment were small and should be interpreted with caution. If there is no such relationship, then this would be consistent with the proposal that top-down resources are not critical for a bias to social information to emerge. In the context of clinical traits, we suggest that individual differences and the underlying cognitive abilities are complex for understanding eye-movement behaviour in scene viewing. One possibility for explaining our finding of fewer fixations on the social object is that ADHD traits may also overlap with ASD. It has been suggested that between 15% and 25% of individuals with ADHD shows ASD symptoms and between 40% and 70% individuals with ASD shows ADHD symptoms (Antshel et al., 2016). Importantly, however; ASD + ADHD is associated with more severe impairments in cognitive and social behaviour when compared with ASD alone (Antshel et al., 2016; Gau & Shang, 2010).

While our study is a step towards understanding the influence of cognitive mechanisms and clinical traits on scene viewing, there are some limitations. First, we examined participants reporting only symptoms of ADHD within undergraduates rather than participants diagnosed with ADHD. Research has shown that individuals who reported high traits of ADHD are likely to report similar impairments than those with the clinical diagnosis (Friedrichs et al., 2012). Also, we assessed ADHD-like symptoms based on the *DSM*-IV criteria. Future studies should assess with questionnaires based on the *Diagnostic and Statistical Manual of Mental Disorders*; 5th ed. (*DSM*-V) criteria which reflect changing knowledge of the symptoms of the disorder. There were also more women in our sample. Research has shown that ADHD is more commonly diagnosed in males compared with females (American Psychiatric Association, 2013). Therefore, future research should place emphasis on the gender differences across adult populations. In addition, as discussed there is evidence from ASD studies showing avoidance of social stimuli. It remains an open question whether our results would be replicated in a clinical sample of ADHD or ASD participants.

In conclusion, we examined the effects of WM and ADHD-like traits on an image-viewing task. Our results suggest that during image-viewing the social object was fixated to a greater degree than the other object across all the conditions. Salience biased our visual attention (regardless of memory loads). However, WM does not seem to affect overall social prioritisation. The relationship between the degree of ADHD-like traits and scanning behaviour was small and only detected on the number of fixations to the social object in the high-salient, low-load condition. Such findings suggest that attending to a social area in complex stimuli is surprisingly not dependent on the availability of default voluntary top-down resources.
